# Evaluation of a self-monitoring protocol for assessing soot and polycyclic aromatic hydrocarbon exposure among chimney sweeps

**DOI:** 10.3389/fepid.2024.1436812

**Published:** 2024-09-04

**Authors:** Therese Klang, Peter Molnár, Christian Lindh, Tobias Storsjö, Håkan Tinnerberg

**Affiliations:** ^1^ Occupational and Environmental Medicine, School of Public Health and Community Medicine, Institute of Medicine, University of Gothenburg, Gothenburg, Sweden; ^2^ Occupational and Environmental Medicine, Sahlgrenska University Hospital, Gothenburg, Sweden; ^3^ Division of Occupational and Environmental Medicine, Lund University, Lund, Sweden

**Keywords:** exposure, measurement, self-monitoring, black carbon (BC), micro-aethalometer, real-time monitoring, direct reading instrument

## Abstract

Traditional methods for measuring chemical exposure have challenges in terms of obtaining sufficient data; therefore, improved methods for better assessing occupational exposure are needed. One possible approach to mitigate these challenges is to use self-monitoring methods such as sensors, diaries, or biomarkers. In the present study, a self-monitored method for measuring soot exposure, which included real-time air monitoring, a work diary, and the collection of urine samples, was evaluated. To validate the method, exposure measurements during the workday and diary entries were compared with velocities calculated from GPS tracking and the expected polycyclic aromatic hydrocarbon (PAH) metabolite patterns in urine. The method was applied with chimney sweeps, an occupational group at a high risk of many severe health outcomes and for whom effective control measures for reducing exposure are needed. In the study, 20 chimney sweeps followed a self-monitoring protocol for 8 consecutive workdays. Personal exposure to soot was measured as black carbon (BC) using micro-aethalometers. A diary was used to record the work tasks performed, and urine samples were collected and analysed for PAH metabolites. From the expected 160 full day measurements, 146 (91%) BC measurements and 149 (93%) diaries were collected. From the expected 320 urine samples, 304 (95%) were collected. The tasks noted in the diaries overlapped with information obtained from the GPS tracking of the chimney sweeps, which covered 96% of the measurement time. The PAH metabolites in urine increased during the work week. Factors believed to have positively influenced the sample collection and task documentation were the highly motivated participants and the continuous presence of trained occupational hygiene professionals during the planning of the study and throughout the measurement stage, during which they were available to inform, instruct, and address questions. In conclusion, the self-monitored protocol used in this study with chimney sweeps is a valuable and valid method that can be used to collect larger numbers of samples. This is especially valuable for occupations in which the employees are working independently and the exposure is difficult to monitor with traditional occupational hygiene methods.

## 1 Introduction

With traditional chemical exposure measurement methods, there are challenges in obtaining a sufficient number of measurements and, therefore, chemical exposure assessments are often inadequate. The difficulty in reaching a sufficient number of measurements is even more pronounced in industries in which the personnel work independently at different locations, as measurements become time-consuming and expensive.

A promising approach to obtain larger numbers of measurements is to use self-monitored measurements (1–3). Studies evaluating and using self-monitoring protocols for exposure measurements are sparse but have been applied for certain exposures, such as benzene (4), terpenes and styrene (2), diesel exhausts (5), volatile organic compounds (VOC) (1), and hydrogen sulphide (6). In the study in which exposure to terpenes and styrene was measured, a comparison was made between self-monitored measurements and measurements performed by professionals. No significant difference in the estimated exposure was found between the two methods, indicating that untrained and unsupervised workers could obtain self-monitored exposure measurements to the same satisfaction as professionals (2).

A sufficient number of measurements or observations are required to perform accurate exposure assessments and determine the variability between the exposed groups, which is known to be important in exposure assessment (7). Factors that have been shown to affect the variability and lead to the need for larger study groups are working outdoors, having intermittent processes, local exhaust ventilation, mobile workers, local sources, and manual working (compared with high levels of automation) (8). Furthermore, repeated measurements are valuable and enable the assessment of within-person and between-person variability (9).

The recent approach to studying the exposome, described as the total exposure of an individual, also establishes a demand for an adequate number of measurements. The exposome is a valuable concept to consider for future epidemiological studies and is one of the driving forces towards the development of sensors (10).

Sensors and real-time monitoring devices have become more frequently used for exposure measurements in occupational and environmental settings. They are often seen as being easier to use, require fewer or no trained professionals or specific handling, require no sample storage pre- and post-measurement, and chemical laboratory analysis of the samples is mostly not needed (11–13). Another valuable factor of many sensors and real-time monitoring devices is the possibility of collecting high-time resolution data. Traditional methods for exposure measurements commonly generate a daily average. With high-time resolution data, it is possible to study the variability throughout the day or during different tasks. This information is important for risk management and making decisions on effective control measures to reduce exposure (11, 14).

Chimney sweeps are an occupational group that work independently and move between locations. They partly work outdoors, have intermittent processes, and primarily perform manual tasks. As they also have an excess risk of many severe health outcomes, they are an important but difficult group to study using traditional means. Notable health outcomes amongst chimney sweeps are an increased risk for developing different cancers (15), myocardial infarction (16), and cardiovascular diseases (17). These severe health outcomes have been connected to the exposure of workers to soot containing polycyclic aromatic hydrocarbons (PAHs) (15). Over the last 35 years, tasks and the use of protective equipment have changed and improved among chimney sweeps in Sweden (18). However, more recent studies have shown that chimney sweeps are still continuously adversely exposed to soot today (17, 19, 20). In recent years, techniques for reducing soot exposure, such as a new sweeping technique called the rotating rod technique, have been developed. With this technique, the opening of the boiler/stove is covered with a plastic cloth that has an opening in it. A vacuum cleaner and a telescopic rod with a sweeping brush at the end are connected to the opening in the plastic cloth. The telescopic rod is connected to a screwdriver, which is used to rotate the brush inside the chimney while the telescopic rod is moving the brush up the chimney. This technique is expected to reduce the exposure of the chimney sweeps to soot, as most of the removed soot is collected inside the covered boiler throughout the sweeping. In addition, the sweep does not have to climb the roof, eliminating the risk of falling down. However, there are also limitations with the technique, one of them being that it cannot be used for all chimney designs due to certain types of pipe bending. To continue reducing occupational exposure, more preventative measures are needed, which requires more information about the exposure. However, an adequate method for studying the exposure of chimney sweeps and other similar working groups has not yet been presented.

There are methods available for assessing the exposure to PAHs in the air and urine, and on the skin (17, 21, 22). These methods are technically advanced and require a trained professional to perform them and interpret the results. Furthermore, they require chemical analysis of the samples. In addition, the trained professional should preferably follow and observe the individuals during the measurement to interpret the results and detect potential deviations occurring during the measurements.

Micro-aethalometers have been previously used to measure soot as black carbon (BC). To our knowledge, measurements of BC have primarily been carried out in urban environments (23, 24), although more sparse undertakings have been conducted in occupational environments (25, 26), and not amongst chimney sweeps. Micro-aethalometers use light transmission technology to measure BC with high-time resolution, which makes it possible to study exposure differences between several working tasks throughout a workday. BC has been used as a proxy for diesel exhausts amongst farmers (25, 26). In this study, we used the method described by Stapleton et al. (26), but as a self-monitoring device used by the participants. In addition to measuring BC and noting work tasks in a diary, participants collected urine samples.

The aim of this study was to evaluate a protocol for the self-monitored measurement of exposure data for chimney sweeps, including measurements of BC in the air, the collection of urine samples, and filling out a work task diary.

## 2 Materials and methods

The protocol for the self-monitored measurement and collection of exposure data described in this study includes measurements of BC in the air, the collection of urine samples, and filling out a work task diary. For the first measurement day for each participant, a more extensive sampling protocol was followed. Over the following 7 days, chimney sweeps performed the monitoring on their own. Only results contributing to the evaluation of the protocol will be presented in this article.

### 2.1 Study group and sampling period

Twenty chimney sweeps were included in the study. Participants were recruited through chimney sweep companies registered with the trade organisation. All interested subjects were informed in detail about the study and the practical aspects of participation. This information was provided by phone or in person. Participation was voluntary and the participants provided written informed consent. The study has been ethically approved by the Swedish ethical review authority (diary number 2020-01218). As data collection was performed at work, participants’ supervisors were also informed and their consent to perform the study during working hours was requested. Initially, companies near the Gothenburg area were recruited, but to recruit enough participants, the search area was extended over a larger geographical area in Sweden.

Exposure data were collected for eight consecutive workdays for each participant (Tuesday of week 1 to Thursday of week 2). No measurements were obtained on Saturday or Sunday. On the first sampling day, a trained occupational hygienist followed each participant throughout the workday to provide instructions, answer questions, observe, and carry out the extended sampling protocol. Each participant received a simplified written manual with pictures of how to handle the device for BC measurements during the self-monitoring period. Over the following 7 measurement days, participants obtained BC measurements, collected urine samples, and filled out a diary by themselves. At the beginning of the self-monitoring period, the occupational hygienist called the participant to enquire whether they had encountered any issues or questions. During the self-monitored measurement period, the occupational hygienist was available by phone. On the last measurement day, the occupational hygienist collected the measuring device, urine samples, and diary. For six chimney sweeps, this was not achievable for practical reasons and the chimney sweeps instead sent the material and samples back by post.

Data were collected between 2020 and 2022, and the chimney sweeps worked primarily with black sweeping using the traditional and/or rotating rod techniques. Chimney sweeping usually includes more tasks than black sweeping, such as fire safety inspections and ventilation control, but in this study the choice was made to only measure during periods of black sweeping.

### 2.2 Micro-aethalometer measurements

BC in the air was measured using micro-aethalometers (microAeth® model MA200 and AE51, AethLabs, San Francisco, CA, USA). Seventeen participants used the MA200 model and three used the AE51 model. Both models use the same technique: the transmission of light at a wavelength of 880 nm is used to determine BC concentration through the change in the particle load collected on a filter (27). The MA200 model can also take measurements at four additional wavelengths that were not used here. Both models have internal flow calibration, which enables a comparison between the set flow and flow during measurement. There is a difference in the filter design between the two models. The MA200 model has a cartridge with filter spots on a tape and as the particle load reaches saturation, the device automatically advances the tape to the next spot and the measurement proceeds. Advancement of the tape will also occur as a new measurement is started. For the AE51 model, the filter needs to be changed manually when saturated by taking it out and replacing it with a new one. Furthermore, the MA200 model includes GPS tracking. In the present study, GPS tracking was used to calculate the worker's speed of displacement (referred to as velocity from here on) between different tasks to validate the diary. This is described in later sections. The measuring range of both models is 0–1,000 µg BC/m^3^ (27). Participants were instructed to start and finish measurements at the beginning and end of each workday and to recharge the device after each workday. The three participants who used the AE51 model were additionally instructed to change the filter after each workday.

Measurements were recorded every 60 s for both models. The micro-aethalometer was placed in a small backpack with the tube mouth placed with a clip in the breathing zone. Personal measurements were taken throughout the workday.

As the loading on the filter spot increases, the result will underestimate the actual BC level according to Kirchstetter and Novakov (28), and a modified correction was performed.

### 2.3 Diary

Participants were instructed to fill out a diary about the work tasks performed during the measurement days. The diary was designed as a questionnaire with boxes to tick for each sweeping task, including the start and finish time of the task, the type of assignment; black sweeping, fire safety inspection/ventilation, ventilation control/cleaning, commercial kitchen/restaurant, the type of object; the resident building, the central boiler or industry, the sweeping technique; traditional or rotating rod technique, fuel, oil, wood, pellet, inside wood fireplace, and other (with the opportunity to write freely). Information on personal protective equipment; the respiratory protection mask, gloves, protective clothes, and/or the vacuum cleaner for the soot was also included. The diary is presented in the Supplementary Material.

### 2.4 Urine samples

Urine samples were collected twice a day, in the morning (first urination of the day) and at the end of the workday, in polypropylene (PP) bottles. Participants were instructed to wash their hands before collecting each sample, label the bottles with the time and date, and store the bottles in the dark and, if possible, in a cold place. Urine samples for each participant were retrieved at the end of the last measurement day. Specific gravity was measured using a manual refractometer. Collected urine was pipetted from the PP bottles into 13-ml PP tubes in duplicate and stored in a freezer, initially at −20°C and later at −80°C due to a shortage of space. As described earlier, for six chimney sweeps, the material, including urine samples, was posted. Owing to logistics, these samples were collected into plastic PP cups, then poured into 13-ml PP tubes and placed into transport tubes by the participants before being sent to the laboratory where they were treated like the other samples. All urine samples were analysed for creatinine by the clinical chemistry laboratory at Sahlgrenska University Hospital, Gothenburg, Sweden, and for the PAH metabolite (1-hydroxypyrene; 1-OH-Pyr) with liquid chromatography coupled to tandem mass spectrometry (LC-MS/MS; QTRAP 5500, AB Sciex, Framingham, MA, USA) at the laboratory of Occupational and Environmental Medicine at Lund University, Sweden, according to Alhamdow et al. (17).

### 2.5 Validation of the protocol

Before the study, a small pilot study with one chimney sweep was performed to evaluate the micro-aethalometers. Sweeping tasks, which were expected to primarily contribute to the soot exposure, were compared with times during which no sweeping tasks were performed (non-task time) to evaluate whether measurements with a micro-aethalometer were relevant for soot exposure. BC levels were higher during sweeping tasks than during non-sweeping times.

**Figure 1:**
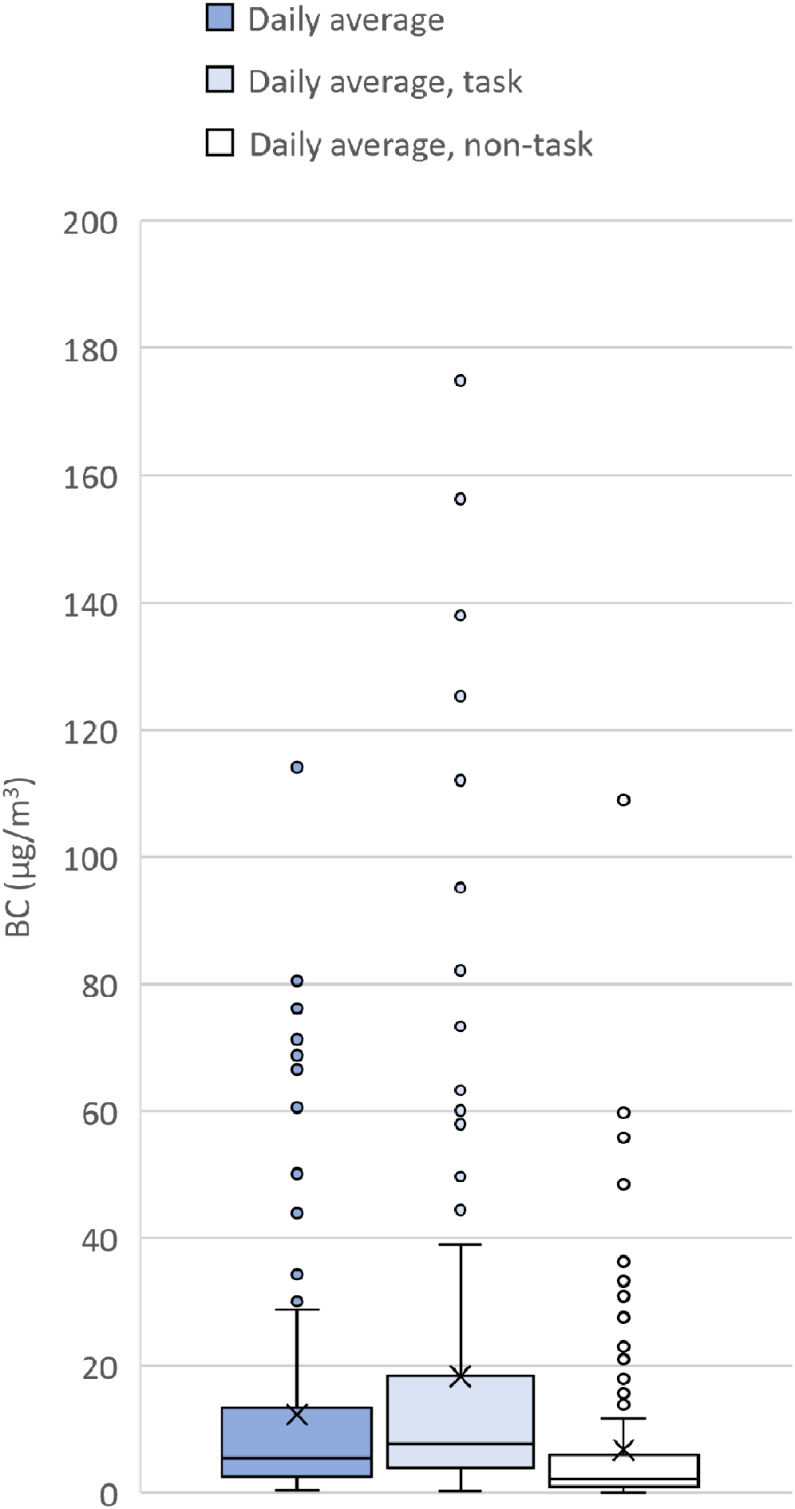
Boxplot of daily average BC levels: all day (sweeping and non-sweeping tasks), sweeping tasks and non-sweeping tasks, respectively. The boxes represent the 25th, 50th and 75th percentiles and whiskers the 1.5 * IQR (inter quartile range) distance from the boxes boundaries. X indicate the arithmetic mean.

In addition in the present study, sweeping tasks were compared with non-task times. This was carried out by comparing the daily mean BC levels in three groups (Figure 1). The first group represented the total daily averages, the second group represented the daily average during times noted in the diaries as task time, and the third group represented the daily averages during non-task time. The groups were compared using paired Student’s *t*-test. Statistical analysis was carried out using SAS software (SAS version 9.4; SAS Institute Inc., Cary, NC, USA), and *p* < 0.05 was considered statistically significant.

Furthermore, it was evaluated how many of the measured data points that were within the instruments measure range of 0–1,000 µg BC/m^3^.

**Figure 2:**
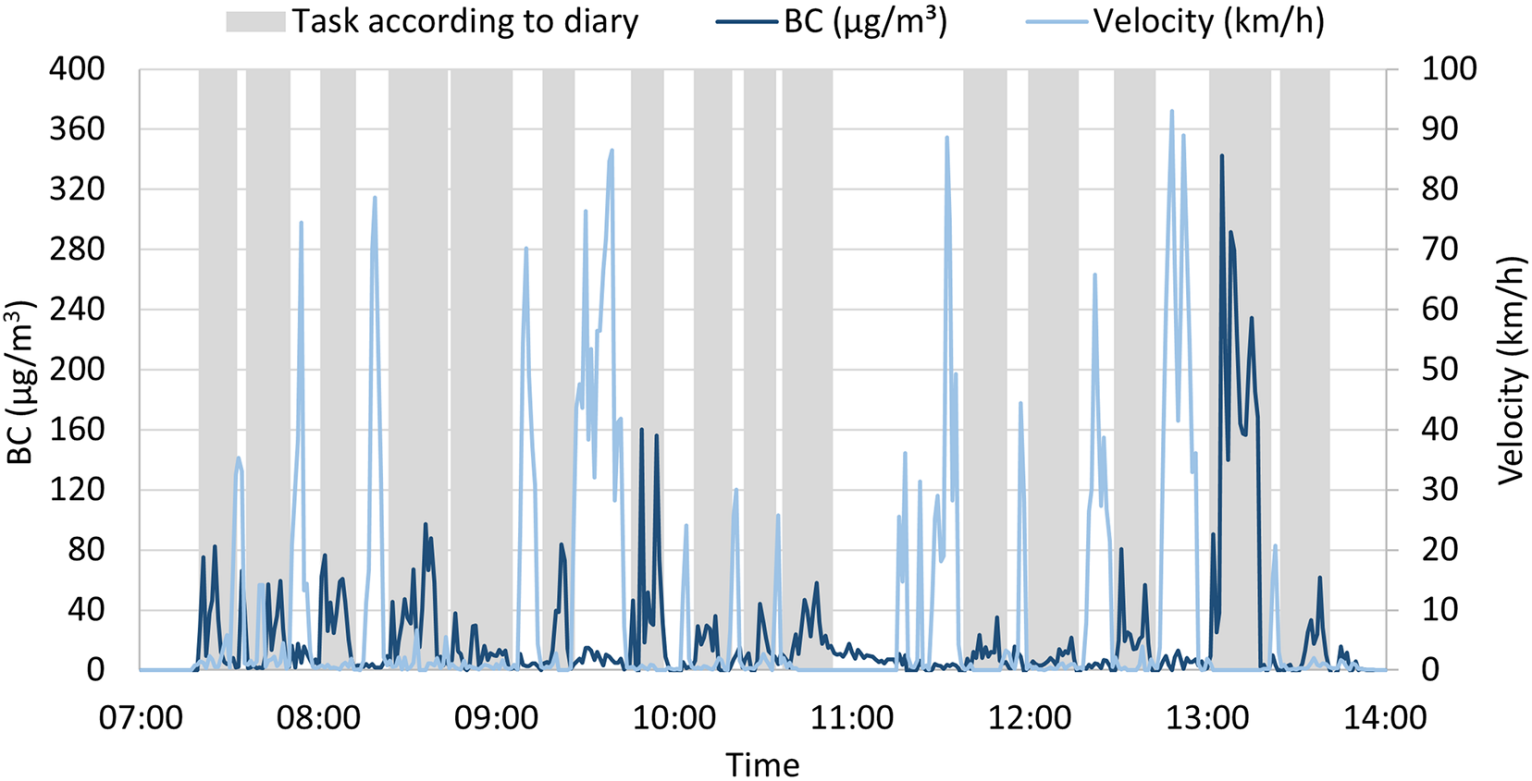
BC level (dark blue), calculated velocity (light blue), and tasks according to the diary (grey) as an example of how they vary and overlap throughout a workday.

The chimney sweeps noted the start and finish times for each task in their diary. From the GPS signal from the MA200 instrument (*n* = 17), we calculated how fast the instrument, and thereby the worker, was moving. By comparing the time periods for the tasks noted in the diary with the calculated velocity, it could be validated how well the chimney sweeps had entered data into the diary. A cutoff velocity of 8 km/h was set to determine transportation by a vehicle and interpreted as periods when a sweeping task would not be possible. This means that time points noted as a sweeping task in the diary for which the velocity was calculated to be 8 km/h or less were interpreted as valid diary entries. An example of diary entries of periods of sweeping tasks, BC levels, calculated velocity, and their overlap is displayed in Figure 2.

**Figure 3:**
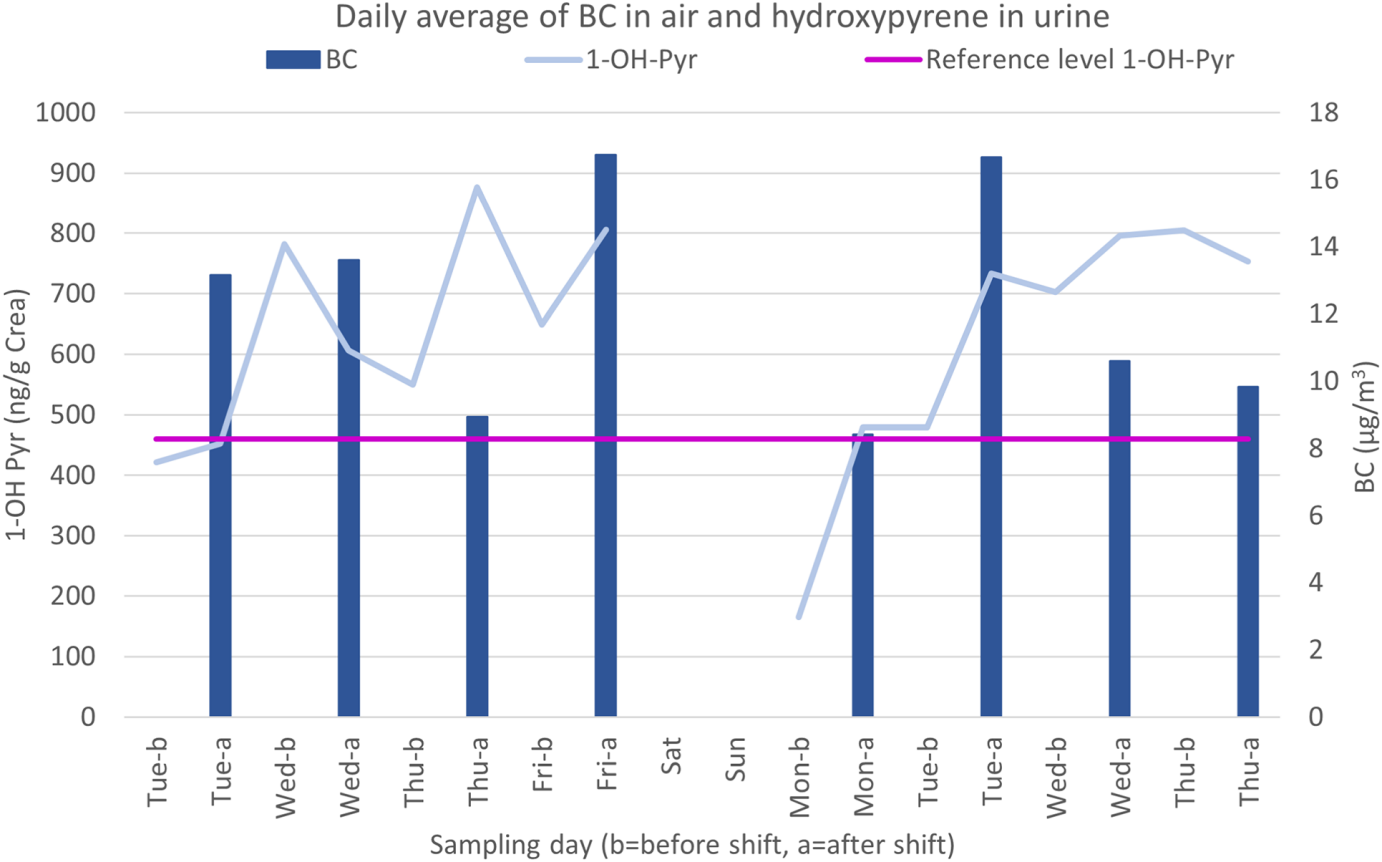
Daily average BC levels in the air and 1-hydroxypyrene (1-OH-Pyr) in urine before (b) and after (a) a shift.

No formal validation was performed for the urine levels of 1-OH-Pyr. However, an informal validation was conducted by displaying the average levels of urine among the chimney sweeps for each day against the average levels of BC (Figure 3).

## 3 Results

### 3.1 Study group and sampling period

The study included 20 chimney sweeps (13 men and 7 women) working with primarily black sweeping during the measurement period. The expected total number of measurement days with the aethalometer, urine sampling, and diary were 160 (8 days for each of the 20 participants).

### 3.2 Micro-aethalometer measurements

In total, 146 micro-aethalometer measurements were collected out of the 160 expected (91%). Of these 146 measurements, 3 were incomplete (1 due to taking care of a child during part of the day and 2 were due to device failure during the day). Of the 14 missing measurements, 6 were due to device failure, 3 were because the participant did not work with black sweeping that day, 2 were due to sick leave or taking care of a child, and 1 was due to a vacation. The cause of the two remaining missing measurements was unknown.

The total study measurement time with the micro-aethalometers was 1,063 h (63,798 min), and the daily mean measurement time was 440 min (241–598 min). Of the 63,798 min of measured BC, 8 min resulted in levels of over 1,000 µg/m^3^, the upper limit of the measuring range of the micro-aethalometers.

The internal calibration of flow rate in both models showed that the flow rate was generally kept within 5% of the set flow. Deviation of the flow rate primarily occurred at the very beginning of a measurement before stabilising.

### 3.3 Diary

Diary entries were recorded on 149 days of the expected 160 days (93%). Of the 11 missing days, 2 were due to micro-aethalometer failure, preventing the participant from filling out the diary, 3 were because the participant did not work with black sweeping that day, 2 were due to sick leave or taking care of a child, and 1 was due to a vacation. For the 3 remaining missing days, the cause was unknown. The total number of sweeping tasks noted in the diaries was 1,545, which accounted for 534 h of the measurement time with the micro-aethalometer (Table 1).

**Table 1:** Collected measurement time.

Total measurement time with the aethalometer (min)	Daily mean measurement time with the aethalometer (min)	Number of tasks noted in the diary	Measurement time with the aethalometer during tasks noted in the diary (min)
63,798^a^ (1,063 h)	440 range: 241–598	1,545	32,051 (534 h)

^a^ Of the 63,798 min, 52,387 min were measured with the MA200 model and the remaining minutes with the AE51 model.

### 3.4 Urine samples

There were 304 collected urine samples out of the 320 expected (95%). Of the 304 samples, 4 were excluded due to an inability to determine the time and date of the sample collection. Of the 16 missing urine samples, 6 were missing due to not working with black sweeping that day, 2 were due to sick leave, 2 due to a vacation, 2 were due to forgetfulness, and the cause was unknown for the remaining 4.

### 3.5 Validation of the protocol

The mean levels of BC (µg/m^3^) are displayed in a boxplot separated into three groups: daily averages for the total measurement time, daily averages only including sweeping tasks, and daily averages only including non-task time (Figure 1). In total, each group includes 145 measurement days. One of the 146 measurement days has been excluded due to missing diary entries for this measurement day. There was a significant difference in the average BC levels between the task time and non-task time groups (12.1 µg/m^3^, 95% confidence interval, 4.0–20.0 µg/m^3^, *p* < 0.0001).

Validation of the diary was performed by looking for overlapping periods between the noted tasks in the diaries and a moving velocity of ≤8 km/h, indicating non-transportation time, calculated with the micro-aethalometer's GPS signal (Figure 2). This validation could only be performed with data from the 17 participants using the MA200 model, as GPS tracking is not available with the AE51 model. The measurement time with the MA200 model was 52,387 min, and of these, 26,289 min were noted as sweeping task time in the diaries. When comparing the time for non-transport (velocity ≤8 km/h) and time for tasks noted in the diary, 25,199 min were interpreted as valid diary entries (96%) (Table 2).

**Table 2:** Validation of the diary against the speed of displacement (velocity) calculated from the GPS coordinates^a^.

Total measurement time with MA200^a^ (min)	Measurement time with MA200 during tasks noted in the diary (min)	Overlap: noted task in the diary and velocity ≤8 km/h (min)	Overlap: noted task in the diary and velocity >8 km/h (min)
52,387	26,289	25,199 (95.9%)	1,090 (4.1%)

^a^ Only measurements obtained with the MA200 model are included as GPS tracking is not available with the AE51 model.

In the figure displaying the average daily BC levels and 1-OH-Pyr levels (Figure 3), the 1-OH-Pyr levels on Monday morning (day 5) are low, as expected, and an increase can be seen during the week. Each measurement point contains, at the least, urine samples from 17 participants. The reference level of 1-OH-Pyr in urine for an unexposed population according to Jongeneelen (29) is 460 ng/g of creatinine and is also included in Figure 3.

## 4 Discussion

In this study, a protocol for the self-monitoring of soot exposure was evaluated. The completion rate for the self-monitoring protocol amongst the chimney sweeps for BC measurements, diary entries, and the collection of urine samples was very high (91%, 93%, and 95%, respectively), with few missing observations. Two specific factors are believed to have promoted the high response rate: the high motivation of the study group and the efforts concerning the information and availability of information before and during the study period. Furthermore, the present research group has studied chimney sweeps for many years, which has produced a well-established network of connections within the industry, enabling effective communication and collaboration during studies.

### 4.1 Study group and sampling period

Only chimney sweeps from the trade organisation were recruited in this study; therefore, there were no participants from companies outside the trade organisation. However, according to the trade organisation, more than 95% of the chimney sweep companies in Sweden are members of the trade organisation.

Owing to logistical reasons, each sampling period started on a Tuesday and finished on a Thursday. This was not optimal. The urine samples collected on Monday mornings are of most concern as these should indicate a baseline for each participant after an unexposed weekend. Owing to the half-life of PAH metabolites, 3.9–35 h (30–32), exposure from Friday is not expected to noticeably affect the levels in urine samples the following Monday morning when compared with unexposed individuals. Figure 3 clearly shows that the levels of PAH metabolites from the Monday morning urine samples are lower than the others and that an increase in metabolites occurs during the week.

There were three missing urine samples for Monday morning, one due to forgetfulness and two due to not working with black sweeping or sick leave. For the missing sample due to forgetfulness, exposure would still be expected that day and therefore no baseline is available. The two later cases resulted in an additional day without an expected exposure and the following workday can instead be seen as a baseline for the measurement period. The same reasoning could be applied for other causes leading to unexposed days. Owing to the half-life time of PAH metabolites, 2 or more consecutive days without exposure are needed to expect similar effects as for a Monday, and no such occasions occurred in the data. Five of the missing urine samples occurred on Fridays during week 1 and were not expected to affect the interpretation of the next sample. Overall, missing samples from measurements with the micro-aethalometer, diary entries, and urine collection generally overlapped.

The intention was to divide the sampling period into two parts: 4 days during which the chimney sweeps used the traditional sweeping technique and 4 days during which the rotating rod technique was used, altering the technique the participants would start with. However, this was not achievable as most participants only used one of the techniques, primarily traditional sweeping.

### 4.2 Micro-aethalometer measurements

Overall, the use of the micro-aethalometer to measure the chimney sweeps soot exposure worked well, as a significant difference between BC levels during task and non-task periods was found, according to the validated diary. This suggests that the micro-aethalometers were suitable for measuring soot exposure. Micro-aethalometers can, in the future, be used by the chimney sweeps, on their own, to increase the understanding of when they are exposed to high levels of soot so that preventive measures can be implemented.

During the project, two different models of micro-aethalometers (MA200 and AE51) were used. For three individuals, the AE51 model was used instead of the MA200 model due to device failure in one case and more parallel measurements being taken than available MA200 devices in the two other cases. The MA200 and AE51 models use the same measurement principle, and there is no reason to suspect a difference in the results between these instruments. The MA200 model includes automatic tape advancement when the filter spot is saturated. This simplifies the measurement for the participant compared with the AE51 model, which requires manual filter changes. When talking to the participants, 19 thought their device was easy to use and they felt confident handling it. The one that did not used an MA200 model and experienced device failure at the beginning of the measurement period, which was solved with technical support from a trained occupational hygienist. The device failure seemed to be unrelated to the handling of the device and the participant completed the measurement period without any further noticeable measurement errors.

To validate the comparability between the two models, side-by-side measurements with several micro-aethalometers were obtained. In the first comparison, one AE51 and two MA200s were situated in a combustion lab at Chalmers University of Technology, Gothenburg, Sweden, where diesel exhausts generated in a controlled fashion were sampled. The BC concentrations were in the range of 2–200 µg/m^3^, with the same instrument setting as for the chimney sweeps. The correlations between the instruments (*r*^2^) were between 0.92 and 0.95, and the slopes were between 0.98 and 1.08. In the other comparison test, four AE51s and two MA200s were placed in a wood boiler room of a single-family home for 24 h. Concentrations were in the range of 0.1–4 µg/m^3^. The correlations between the six instruments were all high (*r* > 0.9).

Despite the variable environment and exposure that the chimney sweeps experience, the internal flow rate calibration of both models showed that the flow was kept within 5% of the set flow. This suggests that both models were suitable for use in this and similar environments.

Overall, the instruments performed well during the measurement campaign. However, one repeated problem with the MA200 model involved the cartridge. For one of the chimney sweeps, this device failure occurred on day 4 and it was not possible to solve the issue for the remaining measurement time. This resulted in four lost measurement days due to this one device failure. Increased instruction to the sweeps on how to solve the problems during the campaign decreased the issues caused by this malfunction.

The measurements were obtained over the whole workday, including lunch breaks, transportation back to the workplace, emptying the vacuum cleaner, and washing their hands back at the workplace. Most chimney sweeps shower and change their clothes at their workplace and measurements were completed before these activities.

Some errors have been found in the result files from the aethalometers. The errors occurred due to device failure or the time change according to daylight savings time during the sampling period, both of which led to mislabelled or missing data in the result files. These minor errors were adjusted manually when found.

In both the pilot study and in the present study, there was a difference in BC levels during the task and non-task periods. In the present study, this difference was evaluated with a statistical test. These results suggest that the micro-aethalometer measurements are relevant for soot exposure.

### 4.3 Diary

The response rate of the diaries (93%) was higher than that in similar studies using diaries (approximately 70%) (33, 34). To validate the task entries in the diary, the velocity of movement calculated from the GPS signal was used together with a cutoff velocity at 8 km/h, which is unlikely to occur during sweeping tasks. Changing the cutoff velocity to 6–10 km/h generated negligible differences (95.2%–96.3% valid diary notes).

Some diary notes could be corrected using the combination of calculated velocity and measured BC levels. One example was where 2 diary entries from the same participant had been noted with the same date. These 2 diary entries could be separated by analysing the overlap between calculated velocity, BC levels and task/non-task periods from the diary, similar to the example in Figure 2.

Ten tasks noted in the diaries had missing start times, 14 had missing finish times, and 5 had missing start and finish times. These 29 tasks are included in the 1,545 tasks described in Table 1. For the 24 tasks with only start or finish times, the task was set to only include the given minute. This means that we underestimated the task time and the difference in BC between the task and non-task time displayed in Figure 1. If it was not obvious how to correct an error noted in a diary, two occupational hygienists discussed how to handle it.

### 4.4 Urine samples

The low number of missing urine samples indicates that the participants were highly motivated to contribute to the study.

In two separate instances, two urine sample bottles had been labelled with the same date and approximately the same time, which meant the samples could not be separated; the four samples were therefore excluded from further analysis.

One concern with the urine samples was the risk of contamination during collection. There was visible soot on the outside of some of the bottles, which was wiped off with damp paper before further handling. It is unclear whether the cause of this soot was due to unclean hands while collecting the sample or whether it occurred later when the bottles were handled. However, contamination during the collection of urine samples would stem from PAHs. The urine samples were analysed for hydroxylated PAHs; a potential contamination of PAHs will not affect the levels of hydroxylated PAHs in the sample, as the hydroxylation of PAHs occurs in cells with the CYP450 enzyme, which is not present in urine.

Exposure levels of 1-OH-Pyr in urine in this study did not differ considerably from an earlier study (17) of PAHs in urine among chimney sweeps. In this study, the median was 0.64 µg/L (0.004–6.4 µg/L), and in the earlier study, the median was 0.56 µg/L (0.03–7.49 µg/L) (adjusted for specific gravity in both cases). When displaying the pattern of excretion of PAH metabolites in urine and the BC levels in the air (Figure 3), it is obvious that PAH levels increase during the working week, which fits with the recommendation of sampling PAH exposure on Thursdays or Fridays. This also clearly indicates the validity of the self-monitoring procedure performed in the study. With biomonitoring through urine samples, as in this study, it is possible to analyse a large number of exposures, such as metals, solvents, pesticides, and organic pollutants, in addition to PAHs.

### 4.5 Limitations

There are limitations to the protocol presented in this study. One limitation is the fact that during self-monitored measurements, there is less control and a lack of observations compared with measurements performed by a trained professional. On the other hand, this is not always achievable during regular hygiene exposure measurements either. However, this affects many different factors, such as noticing and handling possible deviations or method failure appropriately, handling and collecting samples the same way to compare the results, and reporting sufficiently and correctly to enable an adequate interpretation of the results. Examples in the present study include the following: how to place the inlet of the micro-aethalometer tube so that it does not become blocked by clothes; when to collect the urine samples after a shift, as different individuals were different distances away from the facilities where samples could be collected; and when the sweeping task should be noted as finished—is it when you go into the car or when you put down your tools?

Many of these challenges can and have been handled by thorough planning, learning about the field, and providing clear instructions to the participants and having good communication with them. Furthermore, validating with objective measures can and has been undertaken, e.g., GPS tracking, internal time logging by the devices, and assessing whether the levels are realistic. However, despite all efforts to prevent abnormalities, some uncertainty will always remain but it is our belief that the advantages outweigh the limitations when considering self-monitoring with this protocol.

Another area of limitation is the extension of inclusion in our design. Something we chose to exclude was a food diary or food restrictions, which would have been helpful when interpreting the results from the urine samples. This was excluded to lessen the workload of the participants. Instead, to accommodate for the food intake factor, published data from the general population was used. Another limitation is that there are no duplicate samples of BC due to our limited number of devices and the carrying out of parallel measurements of subjects. This would have been valuable for validation. However, as described earlier, side-by-side tests of the devices have been performed to validate the devices.

If possible, we would, of course, have preferred a larger study population and approximately half of the measurement time during each of the sweeping techniques. For the urine samples, it would have been valuable to include the weekend and to have started on the Monday in week one. This would have provided more certainty for the evaluation of the results and also potentially have been easier for the participants as it would be the same procedure every day during their measurement period.

### 4.6 Generalisation

Examples of occupations and environments in which the method could be valuable include environments where exposure to diesel exhausts occur, such as terminals for cargo transport and road workers, as well as environments where combustion occurs, such as the waste industry, the coal and tar production industries, and in foundries. Another expanding occupation in larger cities in Sweden is bicycle couriers; this method could be used to study the exposure to traffic exhausts and correlate the measured concentration levels with the modelled levels using GPS tracking.

### 4.7 Conclusion

The self-monitoring protocol used for the chimney sweeps in this study performed well and is a valuable method, that makes it possible to collect large numbers of samples. The importance of a trained professional in the planning of the study, and who can inform and instruct the participants and be available for questions during the measurement period, was not evaluated, but is probably a significant reason for the high completion rate of this study. Another key factor in the success of self-monitoring measurement is the inclusion of highly motivated participants; however, this was also not evaluated in the study.

The initial analysis of the data indicated a high sample quality, and with this self-monitoring method, using a sensor together with a task-based diary, it is possible to collect large numbers of samples to study exposure to particles and other exposure variables with other independently working personnel. It is concluded that this self-monitoring protocol has been proven to be effective and will be of great value in future studies in aiding the collection of sufficient numbers of samples, especially amongst groups in which individuals work independently.

## Data Availability

The raw data supporting the conclusions of this article will be made available by the authors, without undue reservation.

## References

[1] Brouwer D Keretetse G Nelson G. Quantitative self-assessment of exposure to solvents among formal and informal nail technicians in Johannesburg, South Africa. Int J Environ Res Public Health. (2023) 20(8):5459. 10.3390/ijerph20085459 37107741 PMC10139043

[2] Liljelind IE Rappaport SM Levin JO Strömback AE Sunesson AL Järvholm BG. Comparison of self-assessment and expert assessment of occupational exposure to chemicals. Scand J Work Environ Health. (2001) 27(5):311–7. 10.5271/sjweh.619 11712611

[3] Rappaport SM Lyles RH Kupper LL. An exposure-assessments strategy accounting for within- and between-worker sources of variability. Ann Occup Hyg. (1995) 39(4):469–95. 10.1016/0003-4878(95)00021-6 7661513

[4] Liljelind IE Stromback AE Jarvholm BG Levin JO Strangert BL Sunesson A-LK. Self-assessment of exposure—a pilot study of assessment of exposure to benzene in tank truck drivers. Appl Occup Environ Hyg. (2000) 15(2):195–202. 10.1080/104732200301692 10675977

[5] Hedmer M Wierzbicka A Li H Albin M Tinnerberg H Broberg K. Diesel exhaust exposure assessment among tunnel construction workers-correlations between nitrogen dioxide, respirable elemental carbon, and particle number. Ann Work Expo Health. (2017) 61(5):539–53. 10.1093/annweh/wxx024 28371844

[6] Austigard ÅD Smedbold HT von Hirsch Svendsen K. Risk characteristics of hydrogen sulphide exposure in wastewater collection and treatment related occupations. Ann Work Expo Health. (2023) 67(2):216–27. 10.1093/annweh/wxac065 36124724 PMC9923040

[7] Rappaport SM Weaver M Taylor D Kupper L Susi P. Application of mixed models to assess exposures monitored by construction workers during hot processes. Ann Occup Hyg. (1999) 43(7):457–69. 10.1093/annhyg/43.7.457 10582029

[8] Kromhout H. Design of measurement strategies for workplace exposures. Occup Environ Med. (2002) 59(5):349–54. 10.1136/oem.59.5.349 11983852 PMC1740281

[9] Nieuwenhuijsen M. Exposure Assessment in Occupational and Environmental Epidemiology. New York: Oxford University Press (2003).

[10] Loh M Sarigiannis D Gotti A Karakitsios S Pronk A Kuijpers E How sensors might help define the external exposome. Int J Environ Res Public Health. (2017) 14(4):434. 10.3390/ijerph14040434 28420222 PMC5409635

[11] Goede H Kuijpers E Krone T le Feber M Franken R Fransman W Future prospects of occupational exposure modelling of substances in the context of time-resolved sensor data. Ann Work Expo Health. (2021) 65(3):246–54. 10.1093/annweh/wxaa102 33215191

[12] Ueberham M Schlink U. Wearable sensors for multifactorial personal exposure measurements—a ranking study. Environ Int. (2018) 121(Pt 1):130–8. 10.1016/j.envint.2018.08.057 30199668

[13] Helbig C Ueberham M Becker AM Marquart H Schlink U. Wearable sensors for human environmental exposure in urban settings. Curr Pollut Rep. (2021) 7(3):417–33. 10.1007/s40726-021-00186-4

[14] Sheehan MJ Vosburgh DJH O’Shaughnessy PT Park JH Sotelo C. Direct-reading instruments for aerosols: a review for occupational health and safety professionals part 2: applications. J Occup Environ Hyg. (2022) 19(12):706–29. 10.1080/15459624.2022.2132256 36197433

[15] Hogstedt C Jansson C Hugosson M Tinnerberg H Gustavsson P. Cancer incidence in a cohort of Swedish chimney sweeps, 1958–2006. Am J Public Health. (2013) 103(9):1708–14. 10.2105/AJPH.2012.300860 23327283 PMC3780661

[16] Gustavsson P Jansson C Hogstedt C. Incidence of myocardial infarction in Swedish chimney sweeps 1991–2005: a prospective cohort study. Occup Environ Med. (2013) 70(7):505–7. 10.1136/oemed-2013-101371 23596186

[17] Alhamdow A Lindh C Albin M Gustavsson P Tinnerberg H Broberg K. Early markers of cardiovascular disease are associated with occupational exposure to polycyclic aromatic hydrocarbons. Sci Rep. (2017) 7(1):9426. 10.1038/s41598-017-09956-x 28842704 PMC5573323

[18] Alhamdow A Gustavsson P Rylander L Jakobsson K Tinnerberg H Broberg K. Chimney sweeps in Sweden: a questionnaire-based assessment of long-term changes in work conditions, and current eye and airway symptoms. Int Arch Occup Environ Health. (2017) 90(2):207–16. 10.1007/s00420-016-1186-7 27858151 PMC5263190

[19] Alhamdow A Lindh C Albin M Gustavsson P Tinnerberg H Broberg K. Cardiovascular disease-related serum proteins in workers occupationally exposed to polycyclic aromatic hydrocarbons. Toxicol Sci. (2019) 171:235–46. 10.1093/toxsci/kfz142 31228248 PMC6735884

[20] Alhamdow A Lindh C Hagberg J Graff P Westberg H Krais AM DNA methylation of the cancer-related genes F2rl3 and AHRR is associated with occupational exposure to polycyclic aromatic hydrocarbons. Carcinogenesis. (2018) 39(7):869–78. 10.1093/carcin/bgy059 29722794 PMC6030939

[21] Strandberg B Julander A Sjöström M Lewné M Koca Akdeva H Bigert C. Evaluation of polyurethane foam passive air sampler (PUF) as a tool for occupational PAH measurements. Chemosphere. (2018) 190:35–42. 10.1016/j.chemosphere.2017.09.106 28985535

[22] Kammer R Tinnerberg H Eriksson K. Evaluation of a tape-stripping technique for measuring dermal exposure to pyrene and benzo(a)pyrene. J Environ Monit. (2011) 13(8):2165–71. 10.1039/c1em10245a 21687840

[23] Hofman J Samson R Joosen S Blust R Lenaerts S. Cyclist exposure to black carbon, ultrafine particles and heavy metals: an experimental study along two commuting routes near Antwerp, Belgium. Environ Res. (2018) 164:530–8. 10.1016/j.envres.2018.03.004 29626819

[24] Boniardi L Dons E Campo L Van Poppel M Int Panis L Fustinoni S. Annual, seasonal, and morning rush hour land use regression models for black carbon in a school catchment area of Milan, Italy. Environ Res. (2019) 176:108520. 10.1016/j.envres.2019.06.001 31195294

[25] Sauvé JF Stapleton EM O’Shaughnessy PT Locke SJ Josse PR Altmaier RW Diesel exhaust exposure during farming activities: statistical modeling of continuous black carbon concentrations. Ann Work Expo Health. (2020) 64(5):503–13. 10.1093/annweh/wxaa032 32219300 PMC7313260

[26] Stapleton EM O’Shaughnessy PT Locke SJ Altmaier RW Hofmann JN Beane Freeman LE A task-based analysis of black carbon exposure in Iowa farmers during harvest. J Occup Environ Hyg. (2018) 15(4):293–304. 10.1080/15459624.2017.1422870 29286870 PMC6114936

[27] AethLabs. Aethlabs, Microaeth®/Family (2023). Available online at: https://aethlabs.com/microaeth (Accessed February 16, 2023).

[28] Kirchstetter T Novakov T. Controlled generation of black carbon particles from a diffusion flame and applications in evaluating black carbon measurement methods. Atmos Environ. (2007) 41:1874–88. 10.1016/j.atmosenv.2006.10.067

[29] Jongeneelen FJ. Benchmark guideline for urinary 1-hydroxypyrene as biomarker of occupational exposure to polycyclic aromatic hydrocarbons. Ann Occup Hyg. (2001) 45(1):3–13. 10.1016/S0003-4878(00)00009-0 11137694

[30] Buchet JP Gennart JP Mercado-Calderon F Delavignette JP Cupers L Lauwerys R. Evaluation of exposure to polycyclic aromatic hydrocarbons in a coke production and a graphite electrode manufacturing plant: assessment of urinary excretion of 1-hydroxypyrene as a biological indicator of exposure. Br J Ind Med. (1992) 49(11):761–8. 10.1136/oem.49.11.761 1463676 PMC1039323

[31] Li Z Romanoff L Bartell S Pittman EN Trinidad DA McClean M Excretion profiles and half-lives of ten urinary polycyclic aromatic hydrocarbon metabolites after dietary exposure. Chem Res Toxicol. (2012) 25(7):1452–61. 10.1021/tx300108e 22663094 PMC4618384

[32] Brzeźnicki S Jakubowski M Czerski B. Elimination of 1-hydroxypyrene after human volunteer exposure to polycyclic aromatic hydrocarbons. Int Arch Occup Environ Health. (1997) 70(4):257–60. 10.1007/s004200050216 9342626

[33] Sonnentag S. Work, recovery activities, and individual well-being: a diary study. J Occup Health Psychol. (2001) 6(3):196–210. 10.1037/1076-8998.6.3.196 11482632

[34] Krogh AB Larsson B Salvesen Ø Linde M. A comparison between prospective internet-based and paper diary recordings of headache among adolescents in the general population. Cephalalgia. (2016) 36(4):335–45. 10.1177/0333102415591506 26092285

