# Supplementary material for: Evaluation of a self-monitoring protocol for assessing soot and polycyclic aromatic hydrocarbon exposure among chimney sweeps
Source: Front Epidemiol. 2024 Sep 4;4:1436812. doi: 10.3389/fepid.2024.1436812 (PMC11408179; doi:10.3389/fepid.2024.1436812)
Supplement: Supplementary file 1 [file Datasheet1.pdf]

Supplementary Material

1 Supplementary Data

The diary including work tasks used in the study is displayed in the following pages. The diary has been translated from Swedish to English.

(Fill out once per participant)

Name: ____

Employer/workplace: ____

Study ID (filled out by researcher): __________________________________

Car

Model of car	
Last cleaning of car	

Smoking? Yes ☐ No ☐

(Fill out once per participant)

Time points

<u>Week 1</u> Fill out your working hours for the measurement days (time point for starting and ending the measurement with the device)

	Date	Start (time)	Handling filter in vacuum cleaner (time)	Stop (time)
Measurement day 1				
Measurement day 2				
Measurement day 3				
Measurement day 4				

<u>Week 2</u> Fill out your working hours for the measurement days (time point for starting and ending the measurement with the device)

	Date	Start (time)	Handling filter in vacuum cleaner (time)	Stop (time)
Measurement day 1				
Measurement day 2				
Measurement day 3				
Measurement day 4				

(Fill out for each sweeping task)

Date

Time point when the task

Initiated	*Finished*

1. Type of assignment

	Black sweeping
	Fire safety inspection / Ventilation control / cleaning of ventilation
	Commercial kitchen / Restaurant

Other: ________________________

2. Type of object

	Resident building
	Central boiler
	Industry

3. Sweeping technique

Traditional ☐ Rotating rod ☐

4. Fuel

	Oil
	Wood
	Pellets
	Inside wood fireplace

Other: _____________________

5. Personal protective equipment

	Respiratory protection mask
	Gloves
	Protective clothes<br>(long sleeves and legs on pants?)
	Vacuum cleaner for soot

Filter type:___________________
